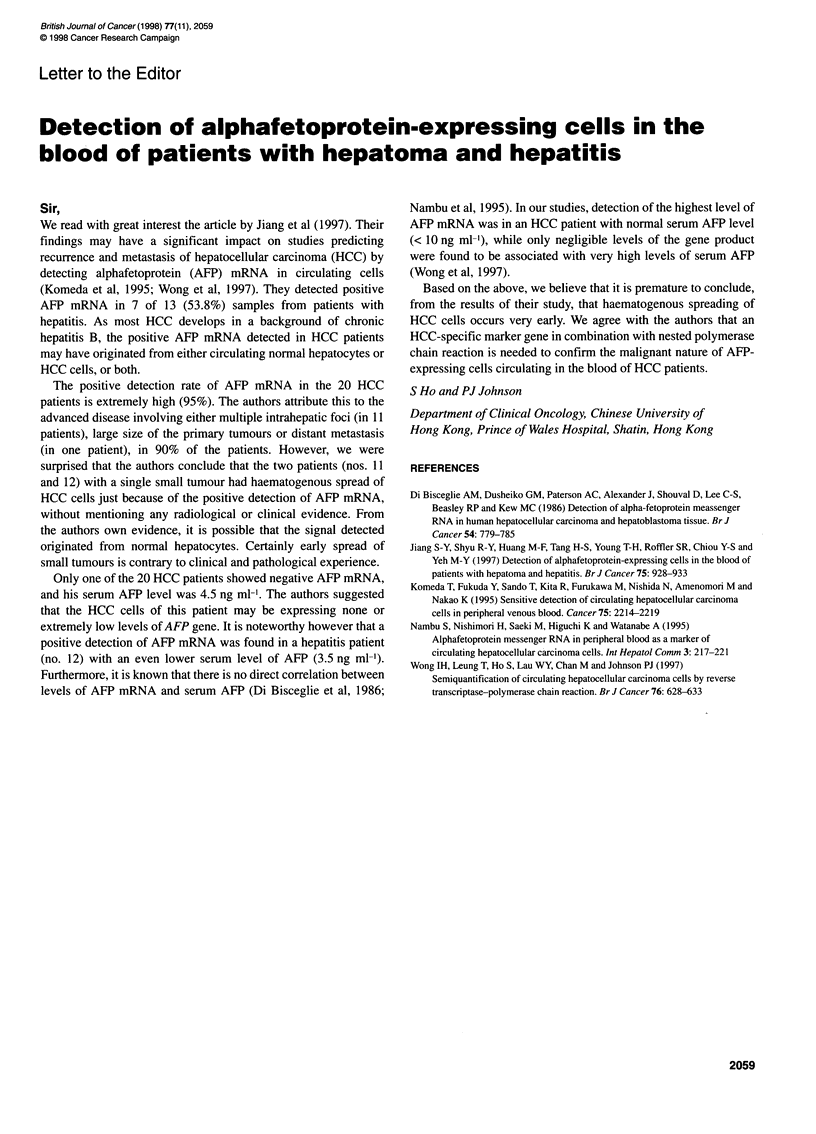# Detection of alphafetoprotein-expressing cells in the blood of patients with hepatoma and hepatitis.

**DOI:** 10.1038/bjc.1998.344

**Published:** 1998-06

**Authors:** S. Ho, P. J. Johnson


British Journal of Cancer (1998) 77(11), 2059
© 1998 Cancer Research Campaign

Letter to the Editor

### Detection of alphafetoprotein-expressing cells in the blood of patients with hepatoma and hepatitis

**Sir,**

We read with great interest the article by Jiang et al (1997). Their findings may have a significant impact on studies predicting recurrence and metastasis of hepatocellular carcinoma (HCC) by detecting alphafetoprotein (AFP) mRNA in circulating cells (Komeda et al, 1995; Wong et al, 1997). They detected positive AFP mRNA in 7 of 13 (53.8%) samples from patients with hepatitis. As most HCC develops in a background of chronic hepatitis B, the positive AFP mRNA detected in HCC patients may have originated from either circulating normal hepatocytes or HCC cells, or both.

The positive detection rate of AFP mRNA in the 20 HCC patients is extremely high (95%). The authors attribute this to the advanced disease involving either multiple intrahepatic foci (in 11 patients), large size of the primary tumours or distant metastasis (in one patient), in 90% of the patients. However, we were surprised that the authors conclude that the two patients (nos. 11 and 12) with a single small tumour had haematogenous spread of HCC cells just because of the positive detection of AFP mRNA, without mentioning any radiological or clinical evidence. From the authors own evidence, it is possible that the signal detected originated from normal hepatocytes. Certainly early spread of small tumours is contrary to clinical and pathological experience.

Only one of the 20 HCC patients showed negative AFP mRNA, and his serum AFP level was 4.5 ng $ml^{-1}$. The authors suggested that the HCC cells of this patient may be expressing none or extremely low levels of *AFP* gene. It is noteworthy however that a positive detection of AFP mRNA was found in a hepatitis patient (no. 12) with an even lower serum level of AFP (3.5 ng $ml^{-1}$). Furthermore, it is known that there is no direct correlation between levels of AFP mRNA and serum AFP (Di Bisceglie et al, 1986; Nambu et al, 1995). In our studies, detection of the highest level of AFP mRNA was in an HCC patient with normal serum AFP level (< 10 ng $ml^{-1}$), while only negligible levels of the gene product were found to be associated with very high levels of serum AFP (Wong et al, 1997).

Based on the above, we believe that it is premature to conclude, from the results of their study, that haematogenous spreading of HCC cells occurs very early. We agree with the authors that an HCC-specific marker gene in combination with nested polymerase chain reaction is needed to confirm the malignant nature of AFP-expressing cells circulating in the blood of HCC patients.

*S Ho and PJ Johnson*

*Department of Clinical Oncology, Chinese University of Hong Kong, Prince of Wales Hospital, Shatin, Hong Kong*

### REFERENCES

Di Bisceglie AM, Dusheiko GM, Paterson AC, Alexander J, Shouval D, Lee C-S, Beasley RP and Kew MC (1986) Detection of alpha-fetoprotein meassenger RNA in human hepatocellular carcinoma and hepatoblastoma tissue. *Br J Cancer* **54**: 779–785

Jiang S-Y, Shyu R-Y, Huang M-F, Tang H-S, Young T-H, Roffler SR, Chiou Y-S and Yeh M-Y (1997) Detection of alphafetoprotein-expressing cells in the blood of patients with hepatoma and hepatitis. *Br J Cancer* **75**: 928–933

Komeda T, Fukuda Y, Sando T, Kita R, Furukawa M, Nishida N, Amenomori M and Nakao K (1995) Sensitive detection of circulating hepatocellular carcinoma cells in peripheral venous blood. *Cancer* **75**: 2214–2219

Nambu S, Nishimori H, Saeki M, Higuchi K and Watanabe A (1995) Alphafetoprotein messenger RNA in peripheral blood as a marker of circulating hepatocellular carcinoma cells. *Int Hepatol Comm* **3**: 217–221

Wong IH, Leung T, Ho S, Lau WY, Chan M and Johnson PJ (1997) Semiquantification of circulating hepatocellular carcinoma cells by reverse transcriptase–polymerase chain reaction. *Br J Cancer* **76**: 628–633